# Oxysterols in catfish skin secretions (*Arius bilineatus*, Val.) exhibit anti-cancer properties

**DOI:** 10.3389/fphar.2022.1001067

**Published:** 2022-10-14

**Authors:** Jassim M. Al-Hassan, Mohammad Afzal, Sosamma Oommen, Yuan Fang Liu, Cecil Pace-Asciak

**Affiliations:** ^1^ Department of Biological Sciences, Faculty of Science, Kuwait University, Kuwait, Kuwait; ^2^ Program in Translational Medicine, Peter Gilgan Centre for Research and Learning (PGCRL), The Hospital for Sick Children, Toronto, ON, Canada; ^3^ Department of Pharmacology, University of Toronto, Toronto, ON, Canada

**Keywords:** epidermal secretions, inflammation, lipids, oxysterols, wound healing, cancer

## Abstract

The edible catfish *Arius bilineatus,* (Valenciennes) elaborates a proteinaceous gel-like material through its epidermis when threatened or injured. Our on-going studies on this gel have shown it to be a complex mixture of several biologically active molecules. Anti-cancer studies on lipid fractions isolated from the gel-like materials showed them to be active against several cancer cell lines. This prompted us to investigate further the lipid composition of the catfish epidermal gel secretions (EGS). Analysis of the lipid fraction of EGS resulted in identification of 12 oxysterols including cholesterol and 2 deoxygenated steroids i.e., 7α-hydroxy cholesterol, 7β-hydroxycholesterol, 5,6 epoxycholesterol, 3β-hydroxycholest-5-ene-7-one and cholesta-3,5-dien-7-one. Progesterone, cholest-3,5-diene, cholesta-2,4-diene, cholest-3,5,6-triol and 4-cholesten-3-one were found as minor components, and were identified through their MS, ^1^HNMR and FTIR spectral data and were compared with those of the standards. Cholest-3,6-dione, cholesta-4,6-diene-3-one, cholesta-2,4-diene, and cholesta-5,20(22)-dien-3-ol were found only in trace amounts and were identified by GC/MS/MS spectral data. Since cholesterol is the major component of EGS, the identified oxysterols (OS) are presumably cholesterol oxidation products. Many of the identified OS are known important biological molecules that play vital physiological role in the producer and recipient organisms. We report herein the effects of these sterols on three human cancer cell lines *in vitro*, i.e., K-562 (CML cell line), MDA MB-231 (estrogen positive breast cancer cell line) and MCF-7 (estrogen negative breast cancer cell line). Interestingly significant (*p* < 0.05) dose differences were observed between tested OS on cell types used. The presence of these sterols in EGS may help explain some aspects of the physiological activities of fraction B (FB) prepared from EGS, such as enhanced wound and diabetic ulcer healing, anti-inflammatory action and cytotoxic activities reported in our previous studies. The anti-proliferative actions of some of these oxysterols especially the cholesterol 3,5,6-triol (#5) as established on selected cancer cell lines in this study support our previous studies and make them candidates for research for human application.

## 1 Introduction

The catfish (*Arius bilineatus,* Valenciennes) was misidentified by fish taxonomists as *Arius thalassinus*, Ruppell. All our publications citing a single catfish species prior to 1988 used their identification (Al-Hassan, et al., 1988). *A. bilineatus,* Valenciennes is an edible catfish consumed by some inhabitants of the Gulf. It elaborates copious amounts of proteinaceous EGS when threatened or injured. This gelatinous material adheres to the fish skin, even though the fish is a fast swimmer and darts in different directions while being towed to the surface on-line.

Fishes with scales on their skin were observed to secrete mucus through their epidermis when they are caught. It was reported that this mucus contains fatty acids, immunoglobulins, lectins, and lysozymes besides glycoproteins (Tiralongo et al., 2020). The mucus was thought to provide skin protection against infection and to help the fish swim fast. However, the catfish A. bilineatus, Val. secretes proteinaceous gel-like secretions when stressed or injured. This epidermal gel secretion (EGS) was found to contain biologically active lipids and proteins (Al-Hassan, et al., 1986; Al-Hassan et al., 1987a; Al-Hassan et al., 1987b). EGS lipid fraction of *A. bilineatus*, Val. contains neutral lipids, glycolipids, and phospholipids. Previous analysis of the lipid fraction indicated the presence of several steroidal compounds (Al-Hassan et al., 1986).

Previously, we postulated that EGS has a protective role for *A. bilineatus* against injury (Al-Hassan et al., 1987b). Results from our laboratory have indicated that protein and lipid content of the EGS display pharmacological activities on blood vessels, blood components, inflamed lesions, cellularity in wound healing in animal model and accelerates diabetic foot ulcer healing in human (Al-Hassan et al., 1983; Al-Hassan, 1990; Al-Hassan et al., 1991), chronic back and joint pain and reduction of inflammation in human (Al-Hassan, 1990; Al-Hassan et al., 2006). Fraction B (FB) containing the catfish lipids was found to kill pancreatic cancer cells, to inhibit CD44 expression and stemness and to alter cancer cell metabolism (Al-Hassan et al., 2021).

Oxysterols can be endogenously produced from cholesterol by enzymatic and/or non-enzymatic oxidative processes or can be absorbed from dietary sources (Ballantine et al., 1980; Vatassery et al., 1997; Carvalho et al., 2010; Luliano, 2011; Chung et al., 2013). It is commonly known that OS show diverse biological activities including cytotoxic (Zmijewski et al., 2009; Carvalho et al., 2011), pro-apoptotic, and pro-inflammatory events (Hwang, 1991; Olkkonen and Hynynen, 2009). In addition, OS, have been implicated in numerous biochemical (Schroepfer, 2000; Bielska et al., 2012; Brown 2012) and pharmacological actions with special emphasis on cellularity (Larsson, et al., 2006; Vejux et al., 2009; Vejux and Lizard, 2009). Recently, it was established that furan fatty acids and Cholesta-3,5-diene (S5, compound #9 in this study), a component of catfish lipids was found to recruit neutrophils and fibroblasts to promote wound healing (Al-Hassan et al., 2020). The anti-inflammatory activity has been attributed to furan fatty acids in the lipid fraction of *Perna Canaliculus* (Murphy et al., 2002; Wakimoto et al., 2011). Cholesta-3,5-diene was found at high level in dog cornea (Cenedella et al., 1992). Fraction B which contains furan fatty acids and oxysterols reduces all pro-inflammatory cytokines and elevates all anti-inflammatory cytokines when injected IP into diabetic animals, as measured in serum after 8 weeks of daily FB treatment (Jassim M. Al-Hassan, unpublished).

An important observation made by one of us (JMH) influenced the direction of this research study. On several occasions, during fishing for catfish for research during the past 46 years, another catfish species (*Arius tenuispenis*, Day) was found to suffer from skin tumors, while *A. bilineatus*, Val. (the subject of this research study) never had such tumors. This raised the question why was this so? This was followed by a determination to investigate EGS for the presence of oxysterols.

Encouraged by our exciting results when FB was applied for treatment of the several health problems cited above, we made it the aim of the present study to focus on the oxysterol content of the lipid fraction of EGS (hence that of FB), as a step towards further understanding the role of each individual OS (tested individually) in the control of inflammation, cancer, diabetes, pain, wound and diabetic ulcer healing, as well as other observed pharmacological activities of FB and its lipid components.

Investigation for oxysterols (OS) and steroids in EGS resulted in identification of twelve oxysterols including cholesterol and two deoxygenated steroids. As EGS was elaborated after shock or injury, the role of each oxysterol and that of the deoxygenated steroids to the fish has yet to be established. The absence of tumors on the skin of *A. bilineatus*, Val. and the discovery of the oxysterols in its EGS encouraged us further to investigate their anti-proliferative activities on selected cancer cell lines.

**FIGURE 1:**
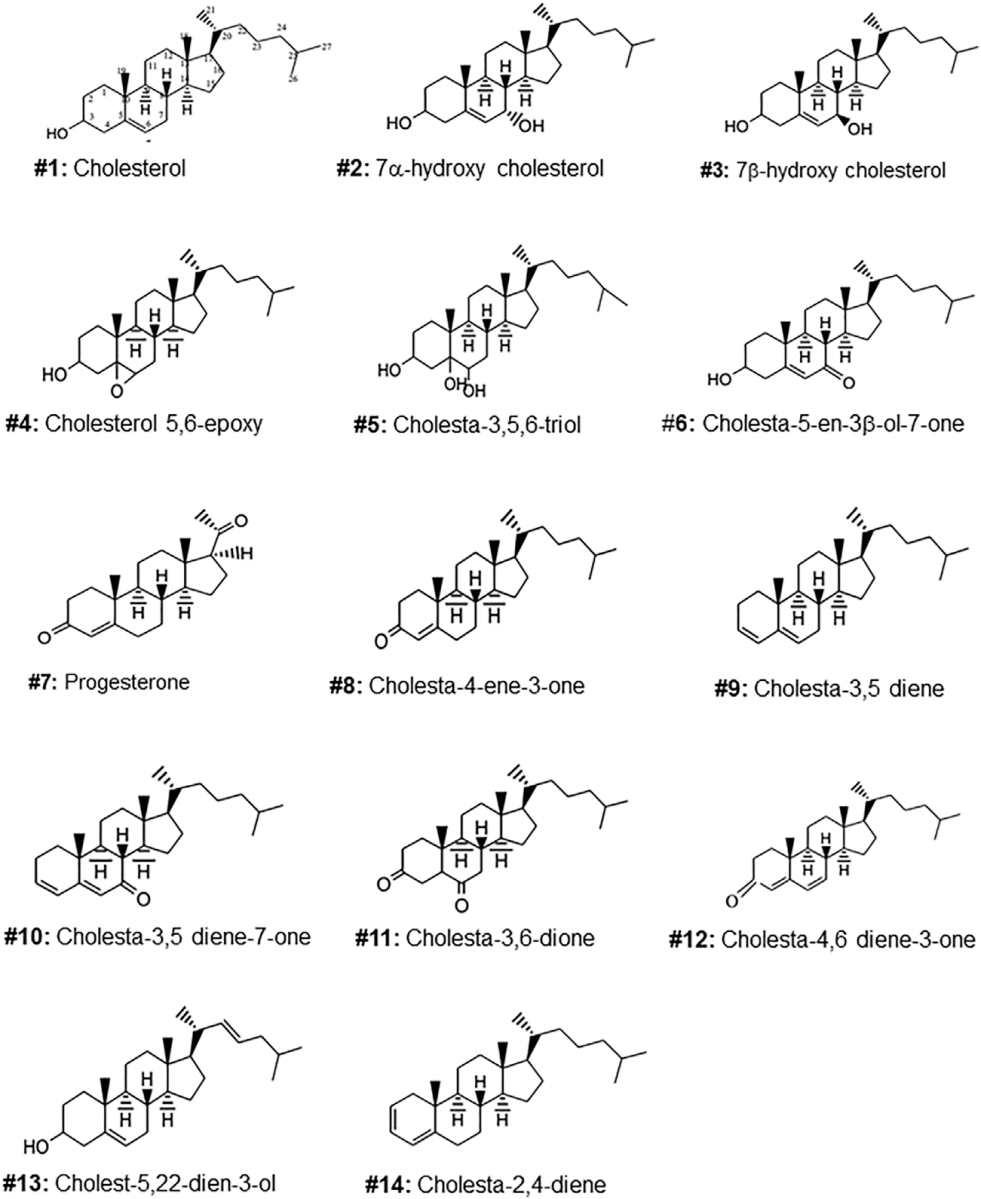
Molecular structures of the steroids isolated from the gel of *Arius bilineatus*. Each structure is assigned with a number to annotate further in the manuscript text.

Since cholesterol is the major steroid component of EGS, the identified oxysterols (OS) are cholesterol oxidation products. Many of the identified OS are known important biological molecules that play vital physiological roles in the producer and recipient organisms (Selley et al., 1996; Staprans et al., 1998; Bjorkhem, 2013). In our study we investigated the effects of these OS on three human cancer cell lines *in vitro*, i.e. K-562 (CML line), MDA MB-231 (estrogen positive breast cancer line) and MCF-7 (breast cancer line estrogen negative). We expect our findings may help to advance new therapies for the active described compounds especially #5 in Figure 1, as supplements to already existing therapies for the specific cancers investigated herein and potentially other cancers.

## 2 Materials and methods

### 2.1 Definition of oxysterols

**FIGURE 2:**
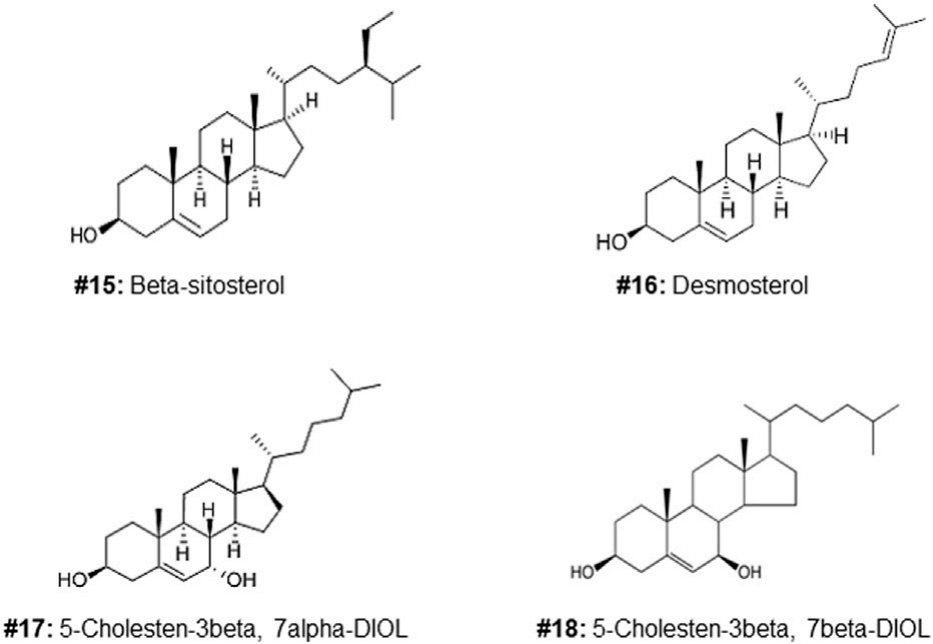
Structures of additional sterols used to investigate the cytotoxic effects of the C5, 6, 7 regions of the molecule (compound #5).

In the subesequent text, OS refers to oxygen (epoxide or keto or hydroxyl)-containing functional groups attached to ring A or B or the side chain of the cholesterol molecule. Several non-oxygenated derivatives of cholesterol (deoxygenated/dehydrated) are referred to as deoxygenated steroids in this nomenclature for convenience of presentation. Cholesterol is a steroid. Structures are shown in Figures 1, 2.

#### 2.1.1 Materials and instrumentation

All analytical grade solvents were freshly redistilled before lipid extraction. Solvents and aluminum coated thin layer chromatography (TLC) plates, coated with fluorescent Kieselgel-60 F_254_, preparative layer chromatography (PLC) and Kieselgel-60 silica gel (0.063–0.100 mm) were obtained from Merck (Darmstadt, Germany). BSTFA + TMCS (99:1, v/v) (Kit # 33154-U) for derivatization of steroids was purchased from Supelco (Bellefonte, PA, United States). Standard steroids, phosphomolybdic acid, and vanillin were obtained from Sigma-Aldrich, United States. Quantification of OS was done using GC/MS/MS spectrometry.

All OS used in the cancer studies *in vitro* were purchased from Sigma-Aldrich, Oakville, ON, Canada) and Steraloids (Newport, RI, United States). RPMI 1640, fetal bovine serum (FBS), antibiotica (penicillin and streptomycin), phosphate buffered saline (PBS) trypan blue and trypsin-ethylenediamine tetra acetic acid (trypsin-EDTAz0 were purchased from Wisent Inc. (St. Bruno, Quebec). Cell lines were purchased from ATCC (Manassas, VA, United States), WST-1 reagent from Sigma-Aldrich, Oakville, ON, Canada, SFM PMI medium from Thermo Fisher Scientific, Waltham, MA, United States.

#### 2.1.2 Analytical instruments and conditions

All spectral measurements including FTIR, ^1^HNMR, ^13^CNMR, GC/MS, and GC-MS/MS were made in Science Analytical Facilities (SAF), Faculty of Science, KU. The GC/MS spectral data were obtained using Agilent-6890 instrument and Trace GC Ultra GC/MS DFS Bremen, Germany. ^1^HNMR, ^13^CNMR data were collected using a 400 MHz NMR (Bruker AC 400, Bruker Co., Switzerland) instrument using deuterated chloroform as a solvent. The analytical conditions for the GC/MS system, used for the analyses of oxysterols were given in Supplementary Table S1.

### 2.2 Skin and muscle tissue sample preparation for lipid extraction

Analysis of the catfish skin and muscle for oxysterols after removal of EGS from the skin was important, considering the popular myth that consuming cooked catfish with its skin intact provides nutritional and pharmacological benefits. To detect oxysterols in skin or muscle of catfish, after removal of EGS, the catfish was repeatedly washed with distilled water accompanied with gentle massaging to ensure removal of any residual EGS. The skin was then carefully removed with a scalpel, homogenized, and freeze-dried. Samples of the flesh immediately under the skin, as well as deep lying flesh, were collected, homogenized, and freeze-dried. Both skin and muscle freeze- dried materials were stored under nitrogen in the dark at −80°C until use. Lipids in the freeze-dried samples were separately extracted using the same lipid extraction procedure as for the EGS material described below.

#### 2.2.1 Preparation of fraction B from catfish epidermal gel secretions

Catfish epidermal gel secretions were collected as described previously (Al-Hassan et al., 1986; ,Al-Hassan et al., 1987b). The gel-like material was fractionated to yield Fraction B (FB), containing lipids (13.4%) and proteins (85%) utilizing procedures described in the two published US patents (Al-Hassan, 2013; Ali 2020). Protein content in FB was assayed with Bradford’s method (Bradford, 1976) employing Coomassie Blue, and the protein concentration was calculated from the standard curve at λ 595 nm generated with bovine albumin. The linear curve obtained was used to calculate total proteins in FB. The lipid percent content of FB was calculated using methods described in (Al-Hassan, 2013; Ali 2020).

#### 2.2.2 Lipid extraction from epidermal gel secretions

Total lipid content of the freeze-dried gel material (5 g) was obtained by extraction with 20–30 ml of chloroform-methanol-isopropanol solvent mixture (2:1:0.25, v/v/v) for 72 h at room temperature under nitrogen environment. The extracted lipids were collected by filtration under vacuum. The remaining solid was re-extracted twice with fresh solvent mixture. The extraction solvent mixture was then pooled and evaporated using a rotary evaporator. The total lipid extract (1.36 g) was re-dissolved in chloroform-methanol (2:1 v/v) solvent mixture for lipid fractionation by silica gel column chromatography.

#### 2.2.3 Fractionation of lipids using column chromatography

The extracted lipid material (1.36 g) was applied to a 30 × 2 cm column packed with silica gel (70–100 mesh, 100–200 μm particle size; Woelm Pharma) in petroleum spirit (40–60°C). The column was first eluted with distilled petroleum spirit (40/60°) to give neutral lipids (NL) (glycerides), followed by a mixture of petroleum spirit:chloroform (1:1 v/v), with a gradual increase of chloroform to 100%, or with petroleum spirit-diethyl ether (8:2, v/v) and 50 ml fractions were collected (we call these fractions also neutral lipid). After TLC analyses of the collected fractions, portions of the same composition were pooled and concentrated on a rotary evaporator. Each concentrated fraction was redissolved in chloroform, passed through a normal phase silica Sep-Pak and analyzed by Agilent GC/MS system.

#### 2.2.4 Purification of oxysterols from the lipid fraction

Analysis for oxysterols in the lipid fractions was carried out using preparative thick layer chromatographic plates (PLC) and thin layer chromatographic plates (TLC). For initial separation of the lipids PLC plates were used. The neutral lipid (petroleum spirit:chloroform column fraction) fraction separated from chromatography column was applied on a PLC plate with the help of an autoliner TLC applicator (Desaga, Sarstedt TLC applicator AS 30, Numbrecht, Germany) and the plate was chromatogrammed in a chromatography solvent chamber containing a mixture of heptane-ethyl acetate (1:1, v/v). After the solvent front reached half the plate, the plate was removed from the chamber and dried under nitrogen environment. The plate was then re-chromatogrammed in a mixture of hexane-diethyl ether-acetic acid (30:19:1, v/v/v). The plate, after drying, was covered with a glass sheet and one side edge of the plate was stained with 1% vanillin solution in 100 ml sulphuric acid-ethanol solution (1:4, v/v) followed by heating the side edge of the plate at 110°C, while keeping the rest of the plate cold. Steroids and oxysterols on the stained part of the PLC plate appeared as a spectrum of different colors. The corresponding unstained bands were scratched off from the PLC plate and the adsorbent was eluted with chloroform:methanol (2:1, v/v). After filtration, the solvent was evaporated under a stream of nitrogen gas and the residue was applied to a TLC plate and chromatogrammed as for the PLC separation above. The side edge of the separated bands was stained with 1% vanillin solution in 100 ml sulphuric acid-ethanol solution (1:4, v/v) followed by heating the side edge of the plate at 110°C. Oxysterols on the stained part of the TLC plate appeared as a spectrum of different colors. The corresponding unstained band of each oxysterol was scratched off from the TLC plate and the adsorbent was eluted with chloroform:methanol (2:1, v/v). After filtration, the solvent was evaporated under a stream of nitrogen gas and the residue was analyzed by GC/MS, FTIR, ^1^HNMR, ^13^CNMR and GC/MS/MS. Spectral data were compared with those of reference spectra. Purity and authenticity of individual compounds were confirmed by TLC and by GC/MS against standards.

#### 2.2.5 Lipid saponification

For confirmation of the type and number of oxysterols in EGS and to make sure that no oxysterols were missed during extraction and purification procedures, saponification was carried out on the total extracted lipids using 3 different methods for comparison purposes. These methods were: 1) Saponification by refluxing in 5 M methanolic NaOH, for 2 h; 2) Reaction with 0.35 N ethanolic KOH for 12 h at 25°C; 3) Incubation in 2 N NaOH mixed with dioxane for 12 h at 25°C. Water (25 ml) was added to the saponification reaction mixture of each procedure, followed by the addition of chloroform (75 ml) and mixed. Dioxane was evaporated after the addition of water under nitrogen. The saponified lipids obtained from each of the saponification procedures were acidified with 2 N HCl and extracted with chloroform. The organic layer was separated, dried over anhydrous sodium sulphate and vacuum evaporated. The residue was then dissolved in chloroform: methanol (2:1; v/v) and one spot from each of the three reactions along with one spot from the neutral lipids were applied to a TLC plate and chromatogrammed in heptane:ethyl acetate (1:1, v:v) mixture till the solvent front reached half the plate. The plate was dried under nitrogen, followed by a second development in hexane:diethylether:acetic acid (30:19:1, v/v/v). The chromatogram was stained with vanillin reagent to detect oxysterols. Samples from the saponifies products were analyzed for OS by GC/MS.

### 2.3 Cytotoxic effects of oxysterols on three human cancer cell lines

K562, MCF-7, MDA MB-231 cells were expanded as per instructions from the Company (ATCC). The cell medium was replaced with fresh RPMI/1% FBS/PS medium. For the studies, the cells were starved for 2 h in a tissue culture flask in SFM RPMI medium. 1e4 cells/well were then added to 96-well plastic plates and conditioned overnight, then centrifuged, replaced with fresh medium with RPMI/1% FBS/PS. The desired amount of each test oxysterol was dissolved in ethanol to make up the concentration needed and was then dried down in a siliconized glass tube then 5 μl ethanol was added, followed by addition of cell medium to make up a concentration of 0.02 μg/μl. The required aliquots were added to each well to which 50 μl conditioned cell medium had been added to make up 100 μl total well volume. The wells were then incubated overnight (<20 h) in 96-well plates. Then 10 μl WST-1 reagent (Roche) was added to each well and incubated for 4 h, then each well was read on a plate reader at 450 nm with 650 nm as reference. Cells were tested for cell viability using trypan blue exclusion method as a routine practice after passaging and counting the cells.

### 2.4 Statistical analysis

GraphPad Prism statistical analysis software (Version 5.0a) was used to perform all statistical analysis. Data are expressed as mean ± standard error. Comparison between multiple groups was performed by ANOVA followed by Bonferroni’s posttest or Dunnett’s test. Pairwise comparisons were performed by *t*-test. A *p*-value of ≤0.05 was considered to represent statistically significant differences between samples.

## 3 Results

### 3.1 Identification of the purified oxysterols from epidermal gel secretion

The TLC purified oxysterols were analyzed by GC/MS, GC/MS/MS, ^1^HNMR, ^13^CNMR and FTIR and their spectral data were compared with those of reference standards (Supplementary Tables S2–S5). The following 13 cholesterol derivatives and cholesterol were identified, and their molecular structures are shown in Figure 1. Cholesterol, 7α-hydroxy cholesterol, 7β-hydroxycholesterol, 5,6 epoxycholesterol, 3β-hydroxycholest-5-ene-7-one, cholest-3,5-diene and cholesta-3,5-dien-7-one were identified by comparing GC/MS/MS, ^1^HNMR, ^13^CNMR and FTIR spectral data with those of reference standards. Progesterone, cholest-3,5,6-triol and 4-cholesten-3-one were found as minor components, and were identified by comparing their MS, ^1^HNMR and FTIR spectral data with those of standards and library data base. Cholest-3,6-dione, cholesta-4,6-diene-3-one, cholesta-2,4-diene, and cholesta-5,20(22)-dien-3-ol were found only in trace amounts and were identified by comparing their GC/MS data with library data base (Supplementary Table S5).

### 3.2 Search for oxysterol in the skin and flesh of the catfish

Analysis of lipids from skin of the catfish, showed the presence of cholesterol and alpha tocopherol with small amounts of free fatty acids and acylglycerols, with no trace of oxysterols. Analysis of the flesh whether located immediately under the skin or deep in muscle layers also showed the presence of only acylglycerols and free fatty acids with a trace amount of tocopherol and cholesterol.

### 3.3 Cytotoxic effects of oxysterols

**FIGURE 3:**
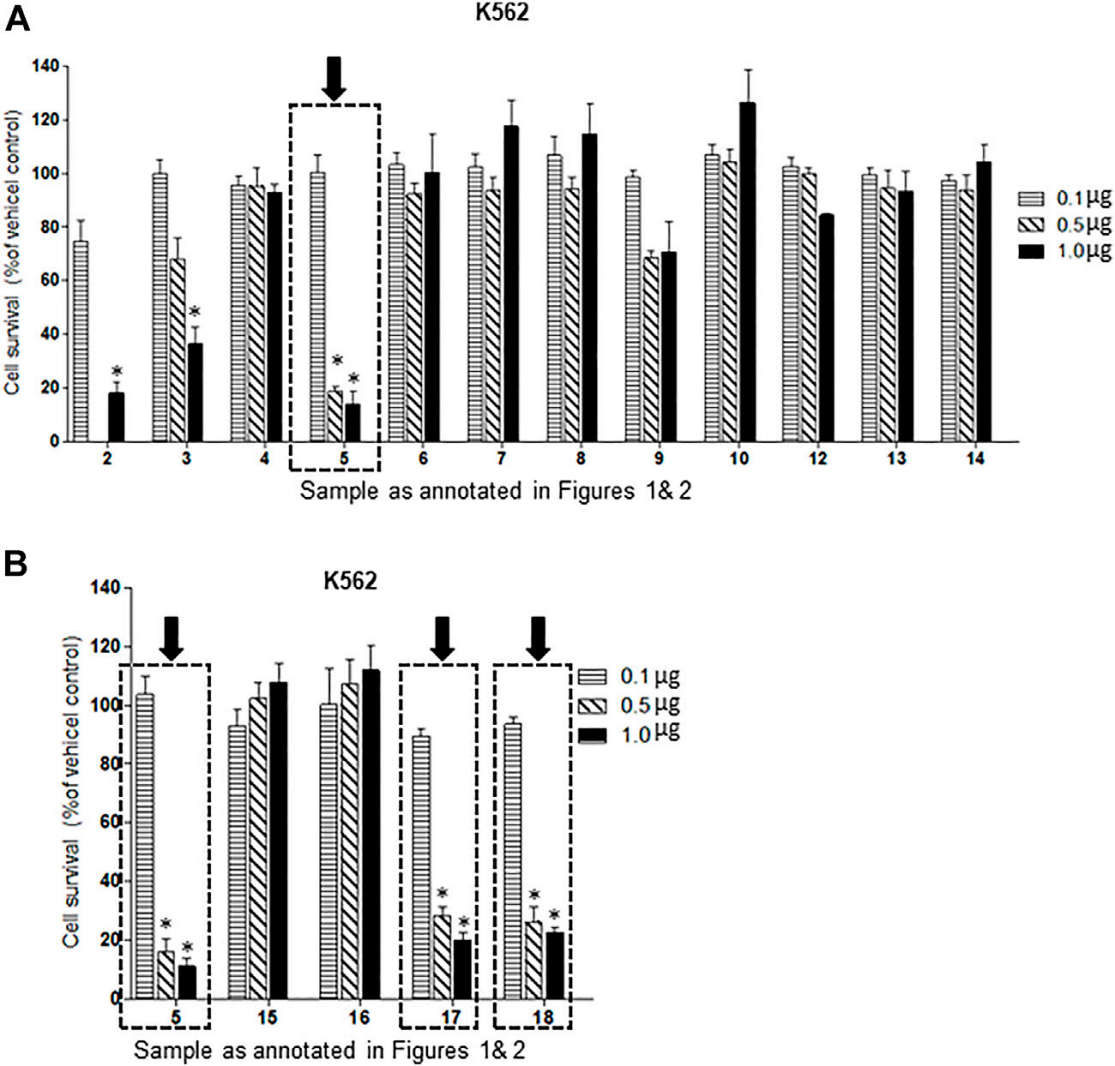
**(A,B)**: Dose-response comparison of the cytotoxic effects of various sterols (as % of ethanol vehicle control) shown to be present in EGS in this study on K562 CML, a human cancer cell line *in vitro*. The cell survival data presented as percentage of vehicle control determine the significant potency of triols #5, #17 and #18 in the tested cell line (*n* = 3, *p* < 0.05, One-way ANOVA with Dunnett’s post-test to compare response of different dosages).

**FIGURE 4:**
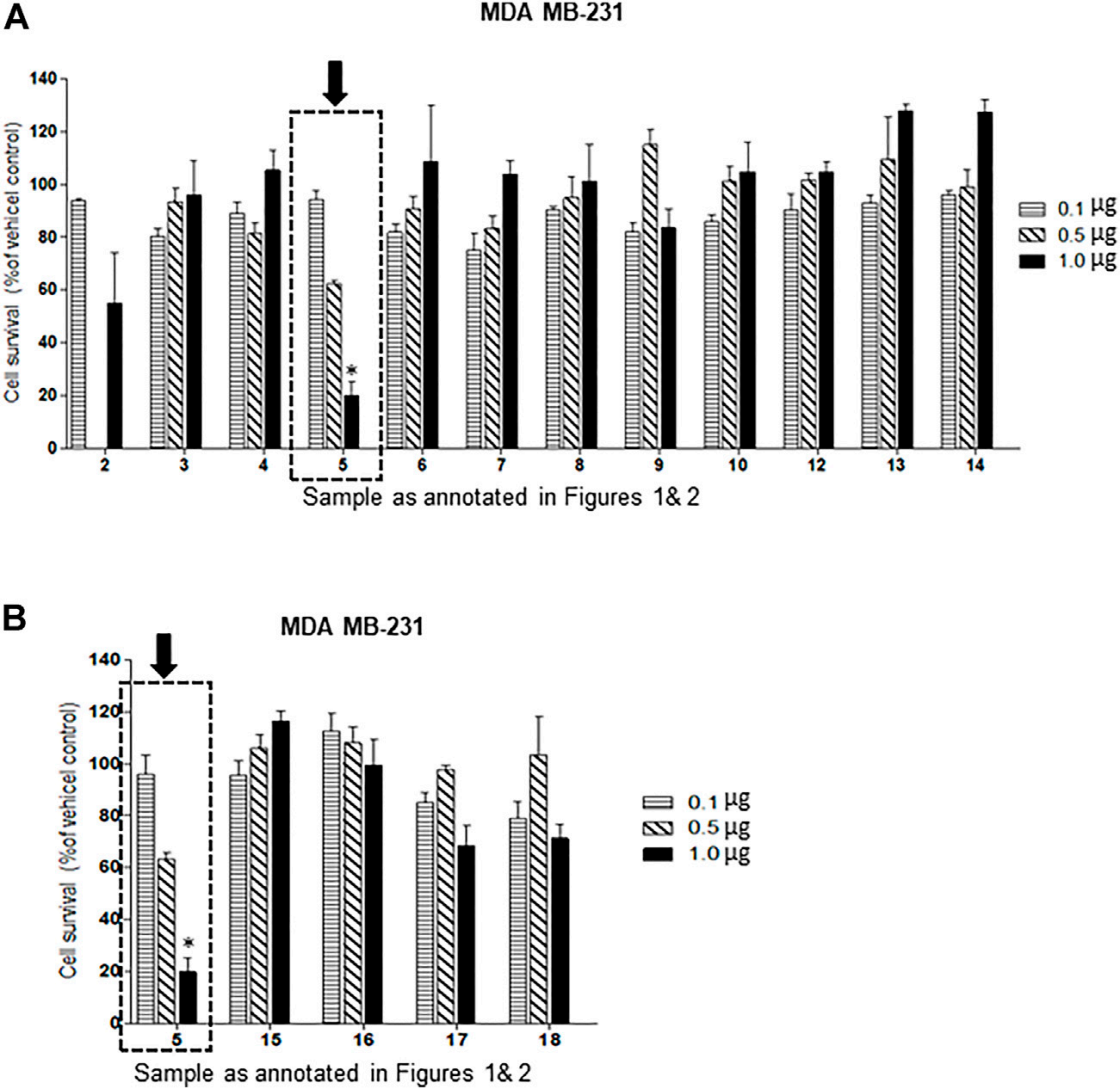
**(A,B)**: Dose-response comparison of the cytotoxic effects of various sterols (as % of ethanol vehicle control) shown to be present in EGS in this study on MDA MB-231 an estrogen receptor negative breast cell line *in vitro*. The cell survival data presented as percentage of vehicle control determine the significant potency of only triol #5 in the tested cell line (*n* = 3, *p* < 0.05, One-way ANOVA with Dunnett’s post-test to compare response of different dosages).

**FIGURE 5:**
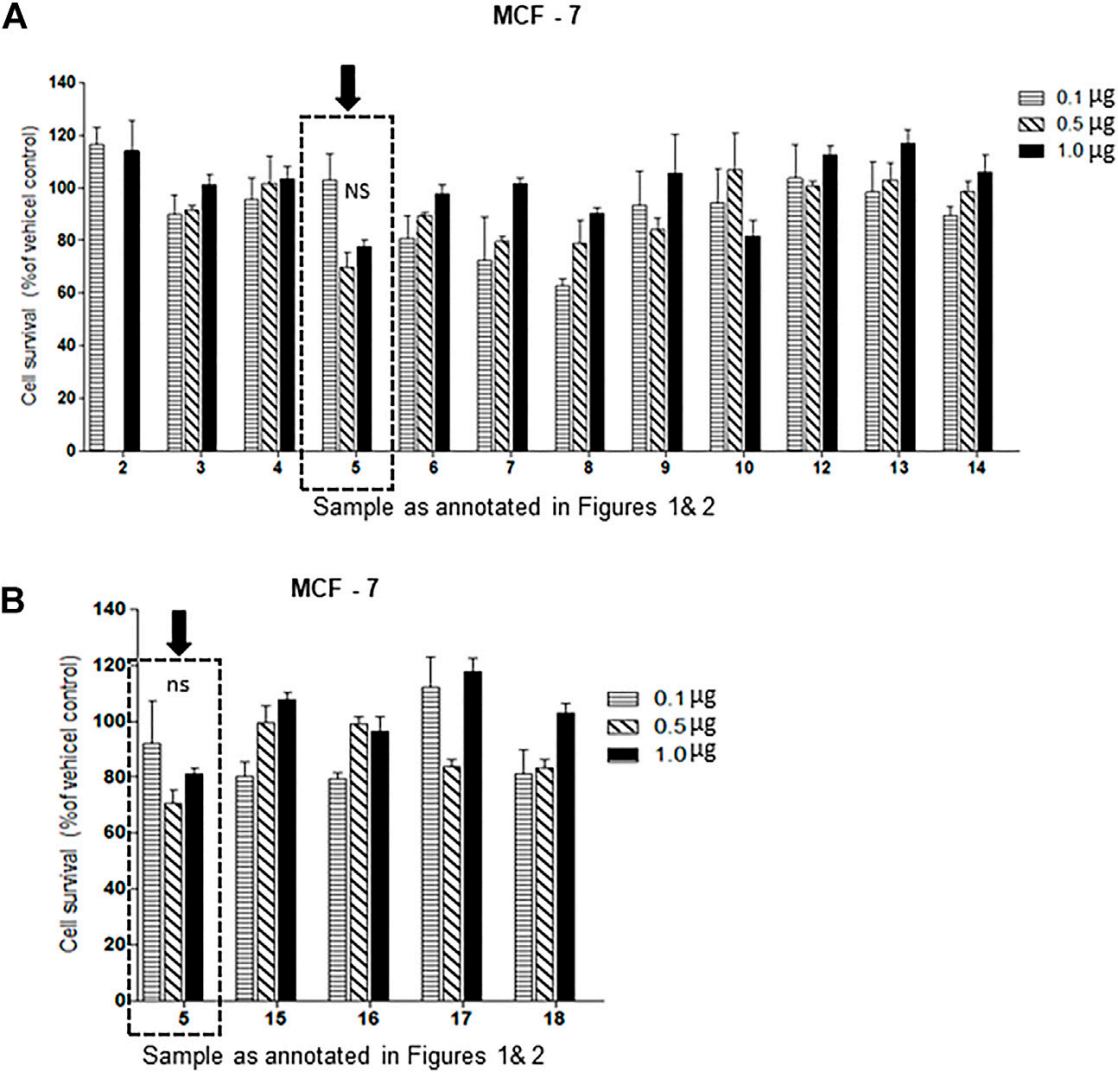
**(A,B)**: Dose-response comparison of the cytotoxic effects of various sterols (as % of ethanol vehicle control) shown to be present in EGS in this study on MCF-7 an estrogen receptor positive breast cell line *in vitro*., Interestingly the cell survival data presented as percentage of vehicle control shows that triols #5 and others are weakly (non-significant) active in MCF-7 cell line (*n* = 3, *p* < 0.05, One-way ANOVA with Dunnett’s post-test to compare response of different dosages).

Of the oxysterol detected in EGS, the most active on all three cell lines was cholesta-3,5,6- triol (#5 in Figure 1) with the K562 leukemic cell line being the most responsive within 0.5–1 μg/100 μl assay volume (Figures 3A,B). Of the two breast cancer cell lines, the MDA MB-231 cell line (Figures 4A,B) was more responsive within this dose range than the MCF-7 cell line (Figures 5A,B). Interestingly the 5,6-epoxide precursor (#4 in Figure 1) of cholesta-3,5,6- triol was not appreciably active, suggesting that the epoxide derives its activity after hydrolysis to the more stable and probably more water soluble triol. Four additional hydroxy cholesterols (#15, 16, 17, 18 in Figure 2) were available commercially which served to investigate the importance of the hydroxyl groups of the 3,5,6 triol in #5 for apoptotic activity, i.e. we compared oxysterols #15–18 with oxysterol #5 (Figures 3B, 4B, 5B). Interestingly, while all 4 oxysterols possessed the 3-hydroxyl group, oxysterols #17 and #18 lacked the 5,6 diol of oxysterol #5, but instead possessed a double bond at C5, 6 as in cholesterol, and a hydroxyl group at C7. Yet #17 and #18 but not #15 and #16 were active as #5 on K562 cells suggesting the importance of C3 hydroxyl group combined with either a C7 hydroxyl group and the double bond at C5,6 or the C5,6 hydroxyl groups could be replaced by a double bond linked with C7 hydroxyl group (Figures 2, 3). The two breast cancer lines were less responsive but still selective for #5 in Figure 1, the cholesta- 3,5,6 triol.

## 4 Discussion

Our studies related to *A. bilineatus*, Val. proved that EGS was composed of biologically active proteins and lipids. This is contrary to the reported composition of fish mucus (Tiralongo et al., 2020). The lipid fraction of EGS was found to be essential for several activities of Fraction B prepared from EGS. Analysis of the lipid fraction indicated the presence of several steroidal compounds. Further search for oxysterols in EGS resulted in twelve oxysterols including cholesterol and two deoxygenated steroids. As it was found that EGS was elaborated after shock or injury, the role of each oxysterol and that of the deoxygenated steroids in the fish during injury or disease has yet to be established.

The presence of oxysterols in the skin of an edible fish has been demonstrated in this study. This will open new venues in fishmeal research with emphasis on pharmacological implications of its oxysteroidal contents. Our results demonstrate that catfish with its skin intact may provide more than nourishing fish proteins and the commonly known fish lipids, such as triglycerides, cholesterol and fatty acids. It can also provide other lipids of biological and pharmacological importance in the neutral lipid fraction as well as glycolipids, and phospholipids.

Healing of wounds and badly infected, highly inflamed non-healing diabetic foot ulcers (some with osteomyelitis) with potent preparations from EGS has been previously reported by us and may be supported by the presence of oxysterols (Al-Hassan et al., 1983; Al-Hassan, 2013; Al-Hassan et al., 2020; Ali 2020). All these activities have been experienced and demonstrated in the EGS and we predict that some of these activities may be correlated with the presence of oxysterols in the epidermal gel material. The available evidence suggests that the androgenic potential of EGS may also be explained based on oxysterols present in the EGS (Wang, et al., 2010).

It was interesting that Cholesta-3,5-diene (cholesterylene; S5, herein labeled #9) which was found in EGS and its FB fraction promotes wound healing (Al-Hassan et al., 2020). The role of each oxysterol found in EGS in the wound and diabetic ulcer healing when FB was applied to these lesions has yet to be established. Analysis of the skin and the flesh of the catfish showed no oxysterols in either of them. The claimed benefits of catfish consumers may be due to incomplete washing of the catfish, leaving some EGS on its skin before cooking, or it could be due to components other than oxysterols in the cooked catfish (anecdotal evidence).

Reports indicate that the OS mixture may result from the free radical oxidation of cholesterol (Bascoul et al., 1986). This is the main lipid component of the fish secreted gel with epoxidation of the C5.6 double bond and its hydrolysis to form cholesterol 3,5,6 triol (#5) a very stable and probably very soluble compound.

We unexpectedly identified interesting dose differences between each OS tested between the three cell types used and between the series of compounds identified. Of these, one in particular (3,5,6-trihydroxy cholesterol metabolite, #5 in Figure 1) was the most potent anti-cancer compound, while its precursor compound, the 3-hydroxy-5,6-epoxide derivative (#4 in Figure 1) was inactive within the doses tested.

The presence of these sterols in EGS may help explain some aspects of the physiological activities of preparations from EGS such as enhanced wound healing and anti-inflammatory and cytotoxic activities reported in our previous studies. These results support our postulate that the skin secretion of catfish may play an important protective role for the fish against injury and disease. The OS which showed anti-cancer activities against selected human cancer cell lines (Figure 3), particularly the potent cholesta-3,5,6 triol, #5 in Figure 1, can be candidates for translational application for curing certain human cancer diseases.

## 5 Conclusion

The presence of twelve OS and two deoxygenated cholesterols in EGS and its FB fraction helps to unravel some of the physiological and therapeutic roles OS provide to the catfish *A. bilineatus*, Val. Our results show that the different OS discovered in EGS may possess different pharmacological activities and that their role could be beneficial to the catfish and may help explain how the catfish heals its wounds and how its skin has not been seen with skin tumors.

The presence of OS together with the other pharmacologically active components found in EGS provides further evidence that EGS is elaborated for the protection of the catfish against possible injuries or skin diseases. The application of FB, which is prepared from EGS for treatment of different human and animal health problems can be considered as an extension to the benefits provided by EGS to the catfish. The action of some OS on selected cancer cell lines reported in this study is exciting. It could inspire other valuable studies based on other active components from the marine environment. Understanding the individual physiological role of each of these OS in EGS may help appreciate their role in our observed wound and diabetic ulcer healing and other physiological activities analogous to those reported here on cancer cells. Thus, results of the present study may lead to correlate various biological activities with the presence of specific OS as shown herein together with the biological activities of the other active molecules found in EGS.

## Data Availability

The raw data supporting the conclusions of this article will be made available by the authors, without undue reservation.
