# Supplementary material for: Oxysterols in catfish skin secretions (Arius bilineatus, Val.) exhibit anti-cancer properties
Source: Front Pharmacol. 2022 Oct 14;13:1001067. doi: 10.3389/fphar.2022.1001067 (PMC9614162; doi:10.3389/fphar.2022.1001067)
Supplement: Supplementary file 1 [file DataSheet1.docx]

**SUPPLEMENTARY MATERIAL**

**­­Oxysterols in catfish skin secretions (Arius bilineatus, Val.) exhibit anti-cancer properties**

**Jassim M. Al-Hassan*^1^, Mohammad Afzal^1^, Sosamma Oommen^1^, Yuan Fang Liu^2^, and Cecil Pace-Asciak^2,3^**

^1^Department of Biological Sciences, Faculty of Science, Kuwait University, P. O. Box 5969, Safat 13060, Kuwait.

^2^Program in Translational Medicine, Peter Gilgan Centre for Research and Learning (PGCRL), The Hospital for Sick Children, Toronto, ON M5G 0A4, Canada

^3^Department of Pharmacology, University of Toronto, Toronto, ON M5S 1A8, Canada

**Running title**: Steroids in catfish (*Arius bilineatus, Val.*) skin secretions show cytotoxic effects

**SM Table 1: Details of GC systems 1 & 2 and their operating program**

| **EQUIPMENT** | **System-1 specifications** | **System-2 specifications** |
| --- | --- | --- |
| GC | Trace GC ultra | Agilent 6890 |
| Detector | GC/MS DFS (Bremen, Germany) | Agilent MSD-5973 |
| Autosampler | TR1 Plus Thermo | Agilent 7683 |
| Liner | Split (P/N: 453T1905) | Split (P/N 5183-4647) |
| Column | TR5MS- 30m× 0.25mm×0.1μm | OV1-30m× 0.25mm×0.1μm |
| **Experimental conditions** | | |
| Inlet Temp | 280^o^ C | 250^o^ C |
| Transfer line | 290^o^ C | 270^o^ C |
| Injection vol. | 1μl | 0.2μl |
| Split ratio | Splitless | 1/50 |
| Carrier gas | Helium | Helium |
| Gas flow | 0.8 ml/min | 1.0ml/min |
| **Oven temperature program** |  |  |
|  | Initial 60^o^ C, 3 min hold; 10^o^ C/min ramp to 250 ^o^C hold 15 min; 10 ^o^C/min ramp to 280 ^o^C, 10 min hold | Initial 70 ^ο^C, 1min hold; 10^o^ C/min ramp to 160 ^o^ C hold 2 min 5 ^o^C/min ramp to 210 ^o^C; 5 min hold 3 ^o^C/min ramp to 250 ^o^ ;15 min hold |
| Software | X-Caliber | Chem station |
| Library | NIST | NIST |

**SM Table 2a**: **^1^H NMR data for standard oxysterols and steroids (values in δ ppm in *d_6_* chloroform).**

| **Compound** | **18-CH_3_** | **19-CH_3_** | **21 CH_3_** | **26, 27 CH_3_** | **CHOH** | **Other significant signals** |
| --- | --- | --- | --- | --- | --- | --- |
| Cholesterol | 1.00 | 0.67 | 0.93 | 0.89 | 3.705 | 5.33 (5C=H) |
| 7α- hydroxycholesterol | 1.02 | 0.71 | 0.92 | 0.88 | 3.69  3.95 | 5.66 (5C=H) |
| 7β-hydroxycholesterol | 1.06 | 0.75 | 0.94 | 0.88 | 3.58  3.88 | 5.32 (5C=H) |
| 5β,6β epoxycholesterol | 1.10 | 0.66 | 0.92 | 0.89 | 3.72 | 3.08 (6-CH) |
| 3β-hydroxycholest-5-ene-7-one | 1.21 | 0.69 | 0.92 | 0.88 | 3.67 | 5.69 (5C=H) |
| Progesterone | 0.65 | 1.19 | 2.13 | --- | ---- | 5.73 (4C=H) |
| Cholest-3,5-diene | 0.97 | 0.72 | 0.93 | 0.88 | ---- | 5.42, 5.63, 5.91, 5.96  (3,5C=H) |
|  |  |  |  |  |  |  |
| Cholest-4,6-diene-7-one | 1.13 | 0.77 | 0.94 | 0.88 | ---- | 5.68, 6.16,6.15, 6.12, 6.10 (4,6C=H) |
| 4-cholest-3-one | 1.19 | 0.72 | 0.93 | 0.88 | ---- | 5.73 (4C=H) |
|  |  |  |  |  |  |  |
| Cholest-3,5-diene-7-one | 1.12 | 0.73 | 0.95 | 0.88 |  | 5.62, 6.01, 6.12, 6.19 (3,5C=H) |

**SM Table 2b:** **^1^H NMR data for oxysterols and steroids isolated from EGS** **(values in δ ppm in** ***d_6_* chloroform).**

| **Compound** | **18-CH_3_** | **19-CH_3_** | **21 CH_3_** | **26,27 CH_3_** | **CHOH** | **Other significant signals** |
| --- | --- | --- | --- | --- | --- | --- |
| Cholesterol | 1.01 | 0.68 | 0.93 | 0.90 | 3.73 | 5.36 (5C=H) |
| 7α- hydroxycholesterol | 1.01 | 0.71 | 0.92 | 0.88 | 3.63  3.89 | 5.60 (5C=H) |
| 7β-hydroxycholesterol | 1.06 | 0.71 | 0.94 | 0.88 | 3.58  3.89 | 5.43 (5C=H) |
| 5β,6β epoxycholesterol | 1.02 | 0.66 | 0.92 | 0.89 | 3.72 | 3.08 (6-CH) |
| 3β-hydroxycholest-5-ene-7-one | 1.20 | 0.75 | 0.94 | 0.89 | 3.63 | 5.67 (5C=H) |
| Progesterone | 0.69 | 1.21 | 2.13 | ---- | ---- | 5.76 (4C=H) |
| Cholest-3,5-diene | 0.98 | 0.72 | 0.94 | 0.88 | ---- | 5.38, 5.42, 5.58, 5.65 (3,5C=H) |
| 4-cholest-3-one | 1.20 | 0.73 | 0.94 | 0.88 | ---- | 5.73 (4C=H) |
| Cholest-3,5-diene-7-one | 1.13 | 0.73 | 0.94 | 0.89 | ---- | 5.62, 6.02, 6.12, 6.20 |

**SM-** **Table 3a:**  **^13^C NMR data for standard oxysterols and steroids (values in ppm in *d_6_* chloroform).**

| **Compound** | **C=O** | **C=H** | **CHOH** | **Epoxy** |
| --- | --- | --- | --- | --- |
| Cholesterol | ---- | 140.8 (5C=H)  121.69 (6C=H) | 71.81 |  |
| 7α- hydroxycholesterol | ---- | 143.38 (5C=H)  124.10 (6C=H) | 71.76  68.71 |  |
| 7β-hydroxycholesterol | ---- | 143.38 (5C=H)  125.45 (6C=H) | 73.37  71.46 |  |
| 5β,6β epoxy cholesterol | ---- | ---- | 68.58 | 65.88 (5C)  59.38 (6C) |
| 3β-hydroxycholest-5-ene-7-one | 202.53 | 165.54 (5C=H)  126.01 (6C=H) | 70.43 |  |
| Cholest-3,5-diene-7-one | 204.92 | 161.06 (5C=H)  124.22 (6C=H)  136.62 (3C=H)  127.72 (4C=H) | --- | ---- |

**SM-** **Table 3b:** **^13^C NMR data for oxysterols and steroids isolated from EGS (values in ppm in *d_6_* chloroform).**

| **Compound** | **C=O** | **C=H** | **CHOH** | **Epoxy** |
| --- | --- | --- | --- | --- |
| Cholesterol | ---- | 140.76 (5C=H)  121.73 (6C=H) | 71.80 |  |
| 7α- hydroxycholesterol | ---- | 143.10 (5C=H)  123.17 (6C=H) | 70.36  69.70 |  |
| 7β-hydroxycholesterol | ---- | 142.89 (5C=H)  125.45 (6C=H) | 73.37  71.46 |  |
| 5β,6β epoxy cholesterol | ---- | ---- | 69.46 | 62.97 (5C)  58.49 (6C) |
| 3β-hydroxycholest-5-ene-7-one | 202.53 | 166.27 (5C=H)  125.66 (6C=H) | 70.07 |  |
| Cholest-3,5-diene-7-one | 204.32 | 163.5 (5C=H)  123.23 (6C=H)  138.15 (3C=H)  127.78 (4C=H) | --- | ---- |

**SM Table 4a: Infrared spectra of standard oxysterols and steroids.**

| **Compound** | **Group Frequencies** | | | |  |
| --- | --- | --- | --- | --- | --- |
|  | **-CO** | **-OH** | **>CH** | **>CH-CH_3_** | **CH=CH** |
| Cholesterol | ----- | 3390.24 | 2933.53 | 2848.67 | 1465.63 |
| 7α- hydroxy cholesterol | ----- | 3326.61 | 2934.01 | 2862.81 | 1462.74 |
| 7β-hydroxy cholesterol | ----- | 3432.67 | 2925.48 | 2860.88 | 1545.67 |
| 5β,6β epoxy cholesterol | 1661.72 | 3406.36 | 2936.4 | 2854.24 | ----- |
| 3β-hydroxy cholest-5-ene-7-one | 1669.80 | 3434.40 | 2949.40 | 2869.10 | 1467.36 |
| Progesterone | 1704.76  1669.09 | ----- | 2934.16 | 2851.24 | 1613.16 |
| Cholest-3,5-diene | ----- | 3438.46 | 2939.95 | 2859.92 | 1643.05 |
| 4-cholest-3-one | 1674.87 | ----- | 2944.77 | 2867.63 | 1613.16 |
| Cholest-3,5-diene-7-one | 1720.90 | ----- | 2950.55 | 2871.49 | 1622.80 |

**SM Table 4b: Infrared spectra of oxysterols and steroids isolated from EGS.**

| **Compound** | **Group Frequencies** | | | | |
| --- | --- | --- | --- | --- | --- |
|  | **-CO** | **-OH** | **>CH** | **>CH-CH3** | **CH=CH** |
| Cholesterol | ----- | 3383.06 | 2943.92 | 2846.75 | 1463.73 |
| 7α- hydroxy cholesterol | ----- | 3436.53 | 2957.30 | 2854.13 | 1570.74 |
| 7β-hydroxy cholesterol | ----- | 3402.78 | 2922.59 | 2852.2 | 1567.84 |
| 5β,6β epoxy cholesterol | 1661.20 | 3427.85 | 2921.63 | 2853.17 | ----- |
| 3β-hydroxy cholest-5-ene-7-one | 1666.38 | 3442.70 | 2948.96 | 2866.02 | 1465.80 |
| Progesterone | 1702.84  1685.48 | ----- | 2947.41 | 2851.24 | 1639.20 |
| Cholest-3,5-diene | ----- | ----- | 2939.95 | 2859.92 | 1643.05 |
| 4-cholest-3-one | 1678.20 | ----- | 2918.73 | 2851.24 | 1632.45 |
| Cholest3,5-diene-7-one | 1737.55 | ----- | 2957.30 | 2854.13 | 1625.80 |

**SM Table 5: Mass spectral data of isolated oxysterols and steroids from EGS.**

| **No.** | **Isolated Compound** | **Mass spectral fragmentation with relative abundance for standard** | **Mass spectral fragmentation with relative abundance for isolated compounds** |
| --- | --- | --- | --- |
| 1 | Cholesterol | M+ 386(100); 368(58) (M+ -H_2_O); 353(47); 301(60); 275(70); 255(30); 213(37); 145(40). | M+ 386(100); 368(50) (M+ -H_2_O); 353(45); 301(57); 275(70); 255(28); 213(35); 145(40). |
| 2 | 7α-hydroxy cholesterol | M+ 402(17); 384(100) (M+ -H_2_O); 366(48)(M+ -2H_2_O ); 351(13); 253(15); 247(18); 211(12); 157(18); 153(29); 143(30). | M+ 402(20); 384(100) (M+ -H_2_O); 366(48) (M+ -2H_2_O); 351(18); 253(10); 247(22); 211(11); 157(18); 153(20); 143(30). |
| 3 | 7β-hydroxy  cholesterol | M+ 402(8); 384(100) (M+ -H_2_O); 366(23) (M+ -2H_2_O); 351(14); 253(9); 247(8); 157(12); 143(12). | M+ 402(5); 384(100) (M+ -H_2_O); 366(20) (M+ -2H_2_O); 351(15); 253(10); 247(8); 157(15); 143(17). |
| 4 | Cholesta-5,6 epoxy | M+ 40 2(100); 384(79); 369(27); 358(24); 331(28); 289(12); 271 (20); 247(25); 163(25); 135 (46); 123(43); 95(62). | M+ 402(100); 384(58); 369(27); 358(25); 331(12); 289(15); 271(20); 247(22); 163(25); 135(52); 123(51); 95(70). |
| 5 | Cholestane-3,5,6-triol | M+ 420(4); 402(100) (M+ -H_2_O); 384(95) (M+ -2H_2_O); 369(54); 331(17); 262(51); 247(63). | M+420(3); 402(100) (M+ -H_2_O); 384(97) (M+ -2H_2_O); 369(48); 331(15); 262(52); 247(70). |
| 6 | Cholest-5-en-3β-ol-7-one | M+ 400(100%), 382(25) (M+ -H_2_O); 367(20)( M+^.^ -H_2_O – CH_3_); 287(15), 192(26), 174(24); 161(27),135(20). | M+ 400(100), 382(23) (M+ -H_2_O); 367(18) (M+ -H_2_O-CH_3_); 287(10); 192 (17), 174(18); 161(19); 135(10). |
| 7 | Progesterone | M+314(95); 299(28) (M+ -CH_3_); 272(85); 229(75); 124(100%); | M+ 314(92); 299(25) (M+ -CH_3_); 272(82); 229(73); 124(100%); |
| 8 | Cholest-4-en-3-one | M+ 384(68); 369(18)(M+ -CH_3_); 342(30); 299(15); 261(40); 229(70); 147(25), 124(100%). | M+ 384(68); 369(18) (M+ -CH_3_); 342(30); 299(15); 261(46); 229(70); 147(28); 124(100%). |
| 9 | Cholesta-3,5-diene | M+ 368(100); 353(33) (M+ -CH_3_); 261(16); 255(17); 247(25); 147(35); 105(20); 81(19). | M+ 368(100); 353(33) (M+ -CH_3_); 261(20); 255(19); 247(29); 147(40); 105(30); 81(26). |
| 10 | Cholesta-3,5-diene-7-one | M+ 382(100); 383(30); 367(15) M+ - CH_3_); 269(18); 187(25); 175(18); 174(99); 161(27); 159(18); 91(20). | M+ 382(100); 383(25); 367(12) (M+ -CH_3_); 269(18); 187(25); 175(19); 174(97); 161(35); 159(22); 91(20) |
| 11 | Cholesta-3,6-dione | M+ 400(100%); 385(20) (M+ -CH_3_); 371(15); 287(32); 260(11); 245(60); 231(23); 137(22); 107(26); 81(34); 55(42). | M+ 400(100%); 385(20) (M+ -CH_3_); 371(17); 287(37); 260(10); 245(70); 231(22); 137(34); 107(28); 81(30); 55(36). |
| 12 | Cholesta-4,6-diene-3-one | M+ 382(100); 367(15) (M+ -CH_3_); 269(43); 247(47); 175(30); 174(30); 160(43); 136(66); 95(35); 81(28). | M+ 382(100); 367(20) (M+ -CH_3_); 269(45); 247(52); 175(30); 174(35); 160(45); 136(68); 95(28); 81(30). |
| 13 | Cholesta-5,22-dien-3-ol | M+ 384(31); 366(25) (M+ -CH_3_); 351(10); 300(40); 271(27); 255(68); 213(36); 159(53); 145(53); 81(70); 69(75); 55(100). | M+ 384(72); 366 (12) (M+ -CH_3_); 351(11); 300(65); 271(45); 255(71); 213(30); 159(69); 145(72); 81(74); 69(90); 55(100). |
| 14 | Cholesta-2,4-diene | M+ 368(100%); 353(25) (M+ -CH_3_); 314(8); 255(40); 213(15); 185(8); 147(35); 105(92); 91(84); 81(51); 55(40). | M+ 368(100); 353(33) (M+ -CH_3_); 314(28); 255(43); 213(26); 185(10); 147(42); 105(78); 91(68); 81(49); 55(50). |

**SM Table 6: Identification of Oxysterols and steroids in Figure 3A-F (Structures are shown in Fig 1, 2)**

| **Assigned numbers for compounds** | **Compound** |
| --- | --- |
| 1 | Cholesterol |
| 2 | 7α-hydroxy-cholesterol |
| 3 | 7β -hydroxycholesterol |
| 4 | Cholesterol-5β,6β-epoxide |
| 5 | 5α -Cholestane-3,5,6-triol |
| 6 | 5-Cholesten-3β -ol-7-one |
| 7 | Progesterone |
| 8 | (+)-4-cholesten-3-one |
| 9 | Cholesta-3,5 diene |
| 10 | Cholesta-3,5 dien-7-one |
| 11 | 5α -cholestane-3,5-dione |
| 12 | 4, 6-cholestadien-3-one |
| 13 | 5, 20(22)-Cholestadien-3β -OL |
| 14 | Cholesta-2,4-diene |
| 15 | β -Sitosterol |
| 16 | Desmosterol |
| 17 | 5-Cholesten-3β, 7α -DIOL |
| 18 | 5-Cholesten-3β, 7β -DIOL |
